# Predictors of high school students’ mathematics self-efficacy in Addis Ababa: The importance of educational expectations

**DOI:** 10.3389/fpsyg.2022.927757

**Published:** 2023-01-06

**Authors:** Yassin Mohammed Yesuf, Sebsibew Atikaw Kebede, Atinkut Zewdu, Dawit Mekonnen Gebru

**Affiliations:** ^1^Department of Psychology, College of Social Sciences and Humanities, University of Gondar, Gondar, Ethiopia; ^2^Department of Mathematics, College of Natural and Computational Sciences, University of Gondar, Gondar, Ethiopia; ^3^Department of Psychology, Institute of Education and Behavioral Sciences, Ambo University, Ambo, Ethiopia; ^4^School of Psychology, College of Education and Behavioral Studies, Addis Ababa University, Addis Ababa, Ethiopia

**Keywords:** mathematics self-efficacy, educational expectations, high school students, Addis Ababa, Ethiopia

## Abstract

In Ethiopia studies on high school students’ mathematics self-efficacy and associated factors are scarce. The present study examined students’ mathematics self-efficacy and associated predictors among high school students in Addis Ababa. Data were collected using adapted questionnaire from 120 students (9th and 10th graders) recruited *via* multi-stage sampling. Descriptive statistics, independent sample *t*-test, ANOVA, Chi-square and logistic regressions were utilized to analyze the collected data. In the study it was found that students have more than average mathematics self-efficacy even though significant numbers of students (44.2%) have low mathematics self-efficacy. It was also revealed that differences in grade level [*t*(118) = 2.545, *p* < 0.05] and students’ expected grade in the upcoming national exam [*F*(3,116) = 5.553, *p* < 0.05] were statistically significant. Living arrangements (AOR = 6.704, 95% CI = 1.598–28.118), expected grade in the upcoming national exam (AOR = 5.808, 95% CI = 1.804–18.696) and expected marks in the semester (AOR = 1.126, 95% CI = 1.055–1.202) are significant predictors of students’ mathematics self-efficacy. Generally educational expectations are important predictors of students’ mathematics self-efficacy. Therefore, researchers and organizations need to gear their attention towards improving students’ mathematics self-efficacy.

## 1. Introduction

The social cognitive theory developed by Albert Bandura is the most prominent learning theory, and self-efficacy is an important component of the theory (Liu and Koirala, 2009). As described by Bandura, self-efficacy is “beliefs in one’s capability to organize and execute the courses of action required to manage prospective situations” (Bandura, 1997). Bandura also described self-efficacy as beliefs about one’s capabilities to learn or perform behaviors at designated levels (Reynolds and Miller, 2003). Heslin and Klehe (2006) argued that self-efficacy is “one of the most powerful motivational predictors of how well a person will perform at almost any endeavor”. A person’s self-efficacy is a strong determinant of their effort, persistence, and strategizing, as well as their subsequent training and job performance (Heslin and Klehe, 2006).

Self-efficacy influences the choices people make and the courses of action they pursue. Most people engage in tasks in which they feel competent and confident and avoid those in which they do not. Beliefs in personal competence also help to determine how much effort people will devote to an activity, how long they will persevere when confronting obstacles, and how resilient they will prove in the face of adverse situations; the higher the sense of efficacy, the greater the effort, persistence, and resilience (Bandura, 1997). Self-efficacy influences the choice we make, the effort we put forth, and how long we persist (Tait–McCutcheon, 2008).

Generally speaking, self-efficacy influences not only an individual’s performance but also the choices they make. The influence of self-efficacy on an individual’s performance and choices is also applicable to students’ performance in school settings. Self-efficacy in an academic setting includes students’ confidence in their cognitive skills to perform the academic task and influences their choice of tasks, persistence, effort, and achievement (Reynolds and Miller, 2003).

Prior national and international studies consistently found that students with high self-efficacy outperform students with low self-efficacy (Huang, 2013; Shaine, 2015; Tizazu and Ambaye, 2017). Moreover, studies depict that self-efficacy determines students’ career decision-making (Ogutu et al., 2017; Akter et al., 2018).

At present, the focus of both educators and policymakers has shifted to science education since a nation’s success is dependent on scientific innovations and advances in technology (Kahveci, 2010). The Ethiopian Ministry of Education, as ratified in Education Sector Development Program V (2015/16–2019/20) has given high priority to science education (Ministry of Education, 2015).

For this to be a reality, students need to join the science and technology fields. But the question here is, “what are the factors that determine students’ future career choices?” Studies conducted to understand what really determines students’ future career choices in science, technology, engineering, and mathematics (STEM) fields have revealed that “high school mathematics achievement, exposure to mathematics and science courses, and mathematics self-efficacy beliefs all affect students’ intent to major in STEM fields, which in turn influences entrance into STEM majors” (Wang, 2013). Mathematics knowledge and skills are requirements not only for tertiary education but also for further studies (Schulz, 2005). Mathematics is a prerequisite for pursuing higher education in most professions, including all exact sciences, finance, programming, and so on. It allows students to choose from a wide range of vocations with high prospects of academic acceptance, mostly in engineering, the natural sciences, and technology, as well as a significant portion of the social sciences (Davidovitch and Yavich, 2018). This calls for a closer look at teaching and learning of mathematics.

In this regard, mathematics self-efficacy, defined as “a situational assessment of an individual’s confidence in her or his ability to successfully perform or accomplish a particular mathematical task or problem”, has become a prominent construct for research (Kiamanesh et al., 2004).

Mathematics self-efficacy (MSE) is one of the crucial factors in students’ mathematics learning (Roslan and Maat, 2019). It is a stronger predictor of math performance than math anxiety (Pajares and Miller, 1994), previous math experience (Pajares and Kranzler, 1995b), mathematics self-concept, perceived usefulness of mathematics, prior experience with mathematics, or gender (Pajares and Miller, 1994) and influences math performance as strongly as overall mental ability (Pajares and Kranzler, 1995a).

In practice, the roles of students’ mathematics self-efficacy on their achievements have been reported in studies conducted at all education levels. For example, in a study among 5th and 6th graders in Spain, students’ mathematics self-efficacy significantly predicted their mathematics achievement (Rodríguez et al., 2020). Mathematics self-efficacy significantly predicted mathematics achievement in 7th graders in Turkey (Recber et al., 2018). Mathematics self-efficacy significantly predicted mathematics achievement in 10th graders in Bhutan (Norbu and Dukpa, 2021).

In a systematic review of studies, it was found that MSE is an important predictor of high school students’ mathematics achievement (Roslan and Maat, 2019). In two studies in Greece among students from grades 7–11 mathematics self-efficacy significantly predicted mathematics achievement using data from the Program for International Student Assessment (PISA) (Cheema, 2018; Hiller et al., 2021). In a study that included sample students from the United States and China aged 15 years, using data from PISA, mathematics self-efficacy significantly predicted mathematics achievement (Wu, 2016). In a study among Bahirdar University students, MSE was a significant predictor of mathematics performance (Getahun et al., 2016).

Alongside these findings, students’ mathematics self-efficacy is found to be a strong predictor of their choice of mathematics-related courses and majors (Zarch and Kadivar, 2006). Besides, studies among college students consistently revealed that MSE is an important predictor of both their performance and major choice (Lent et al., 2008; Lin et al., 2018).

Hence, assessing students’ mathematics self-efficacy would inform us not only about students’ future performance but also about their future career choice. Such assessments need to be conducted at the high school level. This is particularly important in Ethiopia since students are given the opportunity to choose between social and natural science streams after the completion of their high school years (grades 9 and 10). The present study, therefore, aimed to examine high school students’ mathematics self-efficacy in Addis Ababa.

Cognizant of examining students’ mathematics self-efficacy, there is a need to examine the demographic variability among students. Theoretically, the social cognitive theory argues that demographic variability shapes people’s self-efficacy beliefs (Lin et al., 2018). In practice, the effects of demographic variables on students’ mathematics self-efficacy are still inconclusive. For instance, gender was found to be associated with students’ mathematics self-efficacy, favoring males (Lloyd et al., 2005; Wu, 2016; Recber et al., 2018; Rodríguez et al., 2020; Zander et al., 2020). In a meta-analytic review of studies, males were found to have a higher MSE than their female counterparts (Huang, 2013). On the contrary, no gender differences in MSE were observed in other studies (Ayotola and Adedeji, 2009; Clutts, 2010; Turgut, 2013; Davidovitch and Yavich, 2018; Probst, 2019).

School type is another factor associated with students’ mathematics self-efficacy with variable results. For example, in a study conducted by Zedan and Bitar (2014) in Israel, it was found that the dimensions of the classroom environment explain 50% of the variations in high school students’ mathematics self-efficacy. Likewise, in a study among high school students in Greece using data from PISA, students from private schools tend to have higher mathematics self-efficacy than students from public schools (Cheema, 2018). In contrast, in a study in Turkey, there is no difference in MSE based on school type (Recber et al., 2018).

These and other research findings include extensive examinations of variables associated with students’ mathematics self-efficacy. In Ethiopia, studies that assess the mathematics self-efficacy of high school students are scarce, and the ones that are available (mainly student works) come up with contradictory findings. For example, the study by Abebe (2001) found gender differences favoring males, while the studies by Wubalem (2006) and Ayele and Dadi (2016) found no gender differences in students’ mathematics self-efficacy. Contrary to these and many other findings described earlier, a study in Tigray, Ethiopia depicted that female grade 9 students have higher mathematics self-efficacy than their male counterparts (Tekola et al., 2020).

The present study, therefore, tried to examine students’ mathematics self-efficacy and associated predictors among high school students in Addis Ababa. Based on the literature alluded to, we first hypothesized that the students would have a medium level of MSE. It is also hypothesized that there will be variations in students’ MSE based on their demographic characteristics. Besides, students’ demographic characteristics will be important predictors of their MSE. In a country that aspires for attracting a huge amount of students to science and technology fields, a closer inquiry into students’ mathematics self-efficacy would give them the opportunity to make informed decisions. Moreover, the findings of the present study will add new knowledge from a resource poor setting to the inconclusive global findings on the predictive roles of students’ demographic characteristics on their MSE.

## 2. Materials and methods

### 2.1. Research design

The purpose of the present study was to examine high school students’ mathematics self-efficacy and associated factors in Addis Ababa, Ethiopia. For this purpose to be achieved, a quantitative, descriptive, cross-sectional, and explanatory study design was used. The present study is quantitative in terms of the type of data collected. With regard to the timing of the data collection, it is cross-sectional. The study is both descriptive and explanatory in terms of the statistical computations employed to analyze the data.

### 2.2. Sampling technique and sample characteristics

Participants in the study are 120 students selected from two schools in Addis Ababa, Ethiopia. Based on the findings from earlier studies, gender (Rodríguez et al., 2020; Zander et al., 2020), school type (Özgen and Bindak, 2011), and grade level (Wubalem, 2006; Özgen and Bindak, 2011) are important variables in students’ mathematics self-efficacy. As such, an equal number of students from both genders, from both government and private high schools, and from both grades 9 and 10 (60 from each group) were included in the study.

Multistage sampling was used to recruit equal numbers of students from government and private schools. In doing so, first one private and one government school were randomly selected. At the school level, one class from grade 9 and another class from grade 10 were selected again using random sampling. Following that, 15 male and 15 female students were randomly selected from a class (i.e., a total of 30 students were selected from a class). Needless to say, an equal number of students from both genders (60 male and 60 female) and grade levels (60 9th graders and 60 10th graders) were included in the study.

Additional characteristics of the respondents were collected, and summaries of these characteristics are presented in Table 1.

**TABLE 1:** Demographic characteristics of the respondents.

Variables	Category	Mean (SD) or *N* (%)	Min (max)
Age, mean (SD)		16.12 (1.063)	14 (19)
Expected mark, mean (SD)		82.02 (11.514)	45 (100)
Living with, number (%)	One parent	23 (19.2%)	
	Both parents	97 (80.8%)	
Expected grade	A	52 (43.3%)	
	B	58 (48.3%)	
	C	4 (3.3%)	
	F	6 (5.0%)	
Receiving tutorial	Daily	24 (20.0%)	
	Weekly	55 (45.8%)	
	Seldom	5 (4.2%)	
	Never	36 (30.0%)	
Plan to attend college/university	Definitely	93 (77.5%)	
	Maybe	15 (12.5%)	
	No	12 (10.0%)	
Profession	Social science	7 (5.8%)	
	STEM	76 (63.3%)	
	Other	37 (30.8%)	

As shown in Table 1, the mean age of the respondents is 16.12 (SD = 1.063), where the minimum and maximum ages are 14 and 19, respectively. The mean expected mark at the end of the semester is 82.02 (SD = 11.514), where 45 and 100 are the minimum and maximum expected marks, respectively. Of all the respondents, 97 (80.8%) of the respondents live with both parents. With regard to the respondents’ expected grade in the upcoming national exam, 58 (48.3%) of the respondents expect B grade, 52 (43.3%) of them expect A grade, 6 (5.0%) of them expect F grade, and 4 of them (3.3%) expect C grade. Of all the respondents, 45.8% of the students received tutorials weekly, 30.0% of them (36 in number) never received tutorials, 20.0% of them (24 in number) received tutorials daily, and the remaining 4.2% of them (5 in number) seldom received tutorials. In terms of their plan to join a college/university, 93 (77.5%) of the respondents have a definite plan, 15 (12.5%) of them have a tentative plan, and the remaining 12 (10.0%) of them have no plan to attend a college/university in the future. Table 1 also depicts that 63.3% of the students aspire to join STEM, while 5.8% of them aspire to join social science professions. The remaining 30.8% of the respondents planned to join other professions.

### 2.3. Instrument

A questionnaire was used to assess students’ mathematics self-efficacy. Ultimately, the questionnaire has two sections, where the first section collects data on students’ demographic characteristics. This includes school type, age, gender, grade level, living conditions with parents, plans to attend college/university, expected grade in the upcoming national examination, marks expected in the semester, receiving tutorial, and professions students aspire to join. The second section of the questionnaire is adapted from an instrument developed by Pajares (1996) and later modified by Johnson (2008) to be used for assessing high school students’ mathematics self-efficacy. The original tool has 39 items. Before collecting the final data, the adapted tool was translated into Amharic, and a pilot study was conducted on 30 students. In the pilot study, the reliability of the tool was found to be 0.919. The deletion of an item increased the reliability of the tool to 0.924. Therefore, the item “I have never been very excited about mathematics” was deleted from the tool. The final data was thus collected with 38 items measuring students’ mathematics self-efficacy. The replies for the items are based on a five-point Likert scale where 1 = Strongly Disagree, 2 = Disagree, 3 = Undecided, 4 = Agree, and 5 = Strongly Agree. During analysis, 13 items (Pajares and Miller, 1994; Pajares and Kranzler, 1995a; Wubalem, 2006; Zarch and Kadivar, 2006; Lent et al., 2008; Clutts, 2010; Zedan and Bitar, 2014; Ministry of Education, 2015; Wu, 2016; Cheema, 2018; Lin et al., 2018; Probst, 2019; Norbu and Dukpa, 2021) were reverse coded. The highest score, then, represents high mathematics self-efficacy.

### 2.4. Procedures

Permission to conduct the study was secured from Addis Ababa University, School of Psychology’s Ethical Review Committee. Then a formal letter was written from the school directed to the schools, requesting cooperation with the researchers. Then the researchers explained the scope and purpose of the study to the school directors, thereby securing their permission. At the individual level, purpose and scope of the study were communicated with participants, and they were assured that all the information that they would give would be kept confidential. Data are collected after each participant signs the consent form. Participation in this study was totally voluntary, and no compensation was offered.

### 2.5. Methods of data analysis

Descriptive statistics, including percentage, number of cases, mean, scores below, and above the mean and standard deviation, were utilized to describe students’ mathematics self-efficacy and demographic variables collected from the students. In addition, an independent sample *t*-test, a one-way ANOVA, and a chi-square test of significance were utilized to check for differences in mathematics self-efficacy based on associated variables. Simple and multiple binary logistic regressions were computed to assess the effects of the predictor variables over the criterion variable (mathematics self-efficacy). All data were analyzed using the Statistical Package for Social Sciences (SPSS), Windows version 23.

## 3. Results

### 3.1. Students’ mathematics self-efficacy

To describe students’ mathematics self-efficacy, descriptive statistical tools including mean, standard deviation, and minimum and maximum scores were computed. As shown in Table 2, the mean score of the respondents’ mathematics self-efficacy is 129.9 (SD = 22.893), where the minimum and maximum values are 52 and 170, respectively. On a scale of five, the expected mean from the 38-item tool is 114 (3 × 38). Therefore, the mean score found here implies that students have higher than average mathematics self-efficacy. In addition, the standard deviation found here tells us that there is a high dispersion among students’ mathematics self-efficacy.

**TABLE 2:** Description of students’ mathematics self-efficacy.

Variable	*M* (SD)	>mean		<mean		Min (max)
		*N*	%	*N*	%	
Mathematics self-efficacy	129.95 (22.893)	67	55.8	53	44.2	52 (170)

Furthermore, students were categorized as having high or low mathematics self-efficacy using the mean score as a cutoff point. Table 2 also shows that 67 (55.8%) of the respondents scored above the mean value, whereas 53 (44.2%) of them scored below the mean value. This informs us that more than half of the respondents have high mathematics self-efficacy.

### 3.2. Predictors of students’ mathematics self-efficacy

Exploring factors associated with students’ mathematics self-efficacy is one of the main objectives of the present study. To achieve this objective, first of all, mean differences in students’ mathematics self-efficacy based on demographic variables were examined using independent sample *t*-tests and ANOVA tests.

Independent sample *t*-tests were used to look into the mean difference in students’ mathematics self-efficacy based on school type, gender, grade level, and living conditions with parents, and the results of the analysis are summarized in Table 3.

**TABLE 3:** Mean difference in students’ mathematics self-efficacy based on school type, gender, grade level, and living arrangements.

Variables	Category	Mean	SD	*t*-values
School type	Government	127.75	19.198	−1.053
	Private	132.15	26.051	
Gender	Male	130.32	24.696	0.175
	Female	129.58	21.139	
Grade level	9	135.15	15.428	2.545*
	10	124.75	27.641	
Living with	Single parent	133.91	14.529	0.923
	Both parents	129.01	24.426	

*Significant at the 0.05 level.

Table 3 shows us that the mean mathematics self-efficacy of respondents from government schools (*M* = 127.75, SD = 19.198) is lesser than their counterparts from private schools (*M* = 132.15, SD = 26.051), but the mean difference is not statistically significant (*t* = −1.053, *p* = 0.294).

Moreover, Table 3 also shows us that the mean mathematics self-efficacy of male respondents (*M* = 130.32, SD = 24.696) is higher than female respondents (*M* = 129.58, SD = 21.139) but not statistically significant (*t* = 0.175, *p* = 0.862). The table also informs us that the mean mathematics self-efficacy of students who are living with single parents (*M* = 133.91, SD = 14.529) is higher than the mean mathematics self-efficacy of students who are living with both parents (*M* = 129.01, SD = 24.426), although the mean difference is not statistically significant (*t* = 0.923, *p* = 0.358).

On the contrary, the mean mathematics self-efficacy of 9th graders (*M* = 135.15, SD = 15.428) is higher than 10th graders (*M* = 124.75 SD = 27.641), and the mean difference is statistically significant (*t* = 2.545, *p* < 0.05).

One-way ANOVA was employed to look into the mean difference in students’ mathematics self-efficacy based on their expected grades in the upcoming national examination, the number of tutorials they received, their plan to join college/university, and the profession they aspire to join. The summaries of the findings are presented in Table 4.

**TABLE 4:** One-way ANOVA.

Variables	Category	Mean	SD	*F*-values
Plan to join college/university	Definitely	132.63	23.893	2.955
	May be	119.93	15.026	
	No	121.67	18.307	
Receive tutorial	Daily	134.08	35.716	0.734
	Weekly	130.69	18.128	
	Seldom	120.00	27.414	
	Never	127.44	17.819	
Expected grade	A	125.21	25.657	5.553*
	B	136.93	17.683	
	C	100.00	1.155	
	F	123.50	25.034	
Profession	Social science	122.43	7.569	1.149
	STEM	132.25	25.748	
	Other	126.65	17.558	

*Significant at the 0.05 level.

As shown in Table 4, students’ expected grades in the upcoming national examination have a statistically significant effect [*F*(3,116) = 5.553, *p* < 0.05] on students’ mathematics self-efficacy.

Tukey’s HSD *post hoc* test for differences in mathematics self-efficacy indicates that there are statistically significant differences between students who expect an A in the upcoming national examination and students who expect a B (*p* < 0.05) and between students who expect a B and students who expect a C in the upcoming national examination (*p* < 0.01).

Specifically, students who expect a B in the upcoming national examination (*M* = 136.93, SD = 17.683) have higher mathematics self-efficacy than students who expect an A (*M* = 125.21, SD = 25.657). Likewise, students who expect B (*M* = 136.93, SD = 17.683) have higher mathematics self-efficacy than students who expect C in the upcoming national examination (*M* = 100.00, SD = 1.155).

On the contrary, the amount of tutorial received [*F*(3,116) = 0.734, *p* = 0.534], students’ plan to join college/university [*F*(2,117) = 2.955, *p* = 0.056], and students’ professional aspiration [*F*(2,117) = 1.149, *p* = 0.321] do not have a statistically significant effect on students’ mathematics self-efficacy.

In addition to all these computations, chi-square tests were used to check the proportion of respondents with high and low mathematics self-efficacy in each category of the demographic variables. The results of the computations are presented in Table 5.

**TABLE 5:** Proportions of students with high and low mathematics self-efficacy.

Variables	Category	<mean		>mean		χ ^2^
		*N*	%	*N*	%	
Gender	Male	24	40.0	36	60.0	0.845
	Female	29	48.3	31	51.7	
Grade level	9	21	35.0	39	65.0	4.089*
	10	32	53.3	28	46.7	
School type	Government	29	48.3	31	51.7	0.845
	Private	24	40.0	36	60.0	
Living with	Single parent	4	17.4	19	82.6	8.272**
	Both parents	49	50.5	48	49.5	
Receive tutorial	Daily	7	29.2	17	70.8	4.967
	Weekly	25	45.5	30	54.5	
	Seldom	4	80.0	1	20.0	
	Never	17	47.2	19	52.8	
Plan to join college/university	Definitely	36	38.7	57	61.3	5.179
	May be	10	66.7	5	33.3	
	No	7	58.3	5	41.7	
Profession	Social science	5	71.4	2	28.6	5.230
	STEM	28	36.8	48	63.2	
	Other	20	54.1	17	45.9	
Expected grade	A	28	53.8	24	46.2	8.738*
	B	18	31.0	40	69.0	
	Other^∧^	7	70.0	3	30.0	

**Significant at the 0.01 level. *Significant at the 0.05 level. ^∧^Since some counts were below 1, this category is formed from students who expect C and F grades.

Table 5 informs us that the number of 9th graders who score above the mean (58.2%) is higher than the number of 10th graders who score above the mean (41.8%). Table 5 also tells us that the number of 9th graders who score below the mean (39.6%) is lesser than the number of 10th graders who score below the mean (60.4%), and these differences in proportions are statistically significant (χ^2^ = 4.089, *df* = 1, *p* < 0.05).

Besides, the table informs us that the differences in the proportion of students’ mathematics self-efficacy based on their living arrangements are statistically significant (χ^2^ = 8.272, *df* = 1, *p* < 0.01). As such, a higher number of students with single parents scored above the mean (82.6%), while a higher number of students who are living with both parents scored below the mean (50.5%).

Finally, Table 5 reveals that differences in mathematics self-efficacy based on students’ expected grades in the upcoming national examination are statistically significant (χ^2^ = 8.738, *df* = 2, *p* < 0.05). Of the students who expect a B in the upcoming national exam, the majority of them scored above the mean (69.0%). Among students who expect an A or other grades, the majority of them scored below the mean (53.8 and 70%, respectively).

For the sake of exploring the factors associated with students’ mathematics self-efficacy, multiple regression was computed. The result of the computation revealed that the variables added explained 13.6% of the variations in students’ mathematics self-efficacy scores, but the model is statistically insignificant [*F*(10, 109) = 1.722, *p* = 0.0.085, *R*^2^ = 0.136].

As described in the “3.1 Students’ mathematics self-efficacy” section, the mean score was used, and students were categorized as having high or low mathematics self-efficacy. Therefore, bivariate logistic regression models were computed to explore the variables associated with students’ mathematics self-efficacy. Summaries of the results of the computations are presented in Table 6.

**TABLE 6:** Bivariate and multivariate logistic regression results of predictor variables.

Variable	Category	Efficacy		OR (CI)	*p*	AOR (CI)	*p*
		Low	High				
Age				0.624	0.013	0.629	0.112
Expected mark				1.043	0.016	1.126	0.000
Gender	Male	24	36	1.403	0.359		
	Female	29	31	1			
School type	Government	29	31	0.713	0.359		
	Private	24	36	1			
Grade level	9	21	39	2.122	0.044	1.113	0.831
	10	32	28	1			
Living with	Single parent	4	19	4.849	0.007	6.704	0.009
	Both parents	49	48	1			
Expected grade	A	28	24	1			
	B	18	40	2.593	0.017	5.808	0.003
	C	4	0	0.000	0.999	0.000	0.999
	F	3	3	1.167	0.858	9.132	0.136
Receive tutorial	Daily	7	17	2.173	0.165		
	Weekly	25	30	1.074	0.869		
	Seldom	4	1	0.224	0.199		
	Never	17	19	1			
Profession	Social science	5	2	0.471	0.402		
	STEM	28	48	2.017	0.085		
	Other	20	17	1			
Plan to join college/university	Definitely	36	57	1			
	May be	10	5	0.316	0.050	1.527	0.641
	No	7	5	0.451	0.201	3.579	0.306

In the simple logistic regression analysis, age, grade level, living arrangements, expected grades in the upcoming national exam, expected marks from mathematics, and aspiration to join university were significant predictors.

In the multiple logistic regression analysis, only living arrangements, expected grades in the upcoming national exam, and expected marks in the semester from mathematics remain significant. Specifically, it was found that a one-point increase in the marks students expect to score in the semester is associated with a 1.13-point increase in their mathematics self-efficacy (AOR = 1.126, 95% CI = 1.055–1.202). Compared to students who expect A in the upcoming national examination, the odds of having high self-efficacy among students who expect B are five times higher (AOR = 5.808, 95% CI = 1.804–18.696). Students from single parents are six times more likely than students from both parents to have high self-efficacy (AOR = 6.704, 95% CI = 1.598–28.118).

## 4. Discussion

The main purpose of the present study was to examine mathematics self-efficacy and associated predictors among high school students in Addis Ababa. In this study, it was found that the overall mathematics self-efficacy of students is higher than average, and more than half of them have high mathematics self-efficacy, implying that our first hypothesis is confirmed. This finding is consistent with findings from other studies elsewhere. For example, in a study in the Philippines among secondary school students, the mean mathematics self-efficacy of the students was reported to be moderately high with a mean score of 3.25 (Laranang and Bondoc, 2020). Similarly, in a study of 10th graders in Bhutan, the students’ MSE score is reported to be average with a score of 3.25 (Norbu and Dukpa, 2021). Likewise, Perez (2013), who conducted a study in Bangkok, Thailand revealed that more than half of the students showed a self-efficacy score higher than the mean score.

Coupled with this, a study among 12th graders in Bale Zone, Ethiopia assessed students’ belief in mathematics education. The study found a mean score of 3.2 and argued that students have a medium level of belief in mathematics education (Demeke et al., 2020).

On the contrary, in a study in Uganda among high school students, it was found that the mean MSE of students was high with a rate of 4.35 on a scale of five (Batiibwe et al., 2020). The difference could be attributed to methodological variations, where the study in Uganda collected data from only 60 students, using 14 self-opinion items to measure mathematics self-efficacy.

So far as our second hypothesis is concerned, the independent sample *t*-tests and the ANOVA tests confirmed that there are variations in students’ MSE based on grade level and their expected grades in the upcoming national exam. Besides, variations in the proportions of students with high and low MSE are observed based on grade level, living conditions with parents, and expected grades in the upcoming national exam.

With regard to predictors associated with students’ MSE, i.e., our third hypothesis, the present study depicts that students’ expected marks in the semester, their expected grades in the upcoming national exam, and living arrangements with parents are important factors.

In our study, a statistically significant difference was found between 9th graders and 10th graders, where the former had higher mathematics self-efficacy than the latter. This finding is similar to the findings of the study by Özgen and Bindak (2011). The study confirmed that self-efficacy beliefs in Mathematics Literacy vary based on grade, with 9th grade students having the highest ML self-efficacy beliefs and 12th grade students having the lowest. Likewise, in a study among elementary school students (grades 4–6), 4th graders were found to have higher mathematics self-efficacy than their 7th grade counterparts (Lloyd et al., 2005).

On the contrary, some studies found that the mathematics self-efficacy beliefs of high school students do not vary based on grade and that grade does not have an effect in explaining the variance in students’ mathematics self-efficacy (Özyürek, 2010; Batiibwe et al., 2020). The difference in the findings can be explained through methodological differences. The study in Uganda found no difference based on grade level, but it was conducted among 60 students, making group comparisons difficult (Batiibwe et al., 2020). Similarly, the focus of the study by Özyürek (2010) is mainly on sources of self-efficacy scale, not actually on self-efficacy *per se* like our study. Another possible explanation for the differences in findings could be attributed to the fact that we include only two grade levels.

An important finding of the present study is that educational expectations (expressed in the form of anticipating high marks in the semester and good grades in the upcoming national examination) are important predictors of students’ mathematics self-efficacy. Students who expect high grades in the upcoming exam and higher marks in the semester were found to have significantly higher mathematics self-efficacy than their counterparts. According to the findings of the present study, self-educational aspiration has been reported to have a positive relationship with students’ MSE in a study among 5th and 6th graders in China (Liu et al., 2020). Similar findings were reported from a study in South Africa among 9th graders (Fadiji and Reddy, 2021).

According to Bandura’s theory, students with high self-efficacy expect success in future tasks. Within the theory, it is claimed that it is individuals’ efficacy that determines their goal-setting (Bandura, 1997). Thus, our findings substantiate Bandura’s claim that self-efficacy and future expectations are related.

One of the interesting findings of this study is the effect of living arrangements with parents on students’ mathematics self-efficacy. In the chi-square test, it is depicted that the majority of the students who are living with single parents have high MSE. Besides, the regression analysis has shown that the odds of students with a single parent having high MSE are six times higher than those of students who are living with both parents.

This finding is interesting in that living with single parents seems to be a contributing factor to students’ mathematics self-efficacy. Given the relatively small numbers of students from single parents in the study (19.2%), this finding needs to be interpreted carefully.

Actually, other studies have indicated other parent-related factors in students’ mathematics self-efficacy. For instance, in a study in the Philippines among high school students, mothers’ occupation, fathers’ occupation, and parental monthly income were related to students’ MSE scores (Laranang and Bondoc, 2020).

Likewise, in a study in Greece using data from PISA among 7–11 graders socioeconomic status (measured by parental education, parental occupation, and an index of home possessions) was associated with students’ MSE (Hiller et al., 2021). In another study that used data from PISA and included sample students from the United States and China, socioeconomic status (measured by parental education, parental occupation, and an index of home possessions) was associated with students’ MSE (Wu, 2016).

Besides, the roles of parental involvement in students’ MSE are reported in prior studies (Howard, 2015; Rodríguez et al., 2017). Although not on MSE, in a study among university students in Ethiopia, parenting style was found to be an important predictor of students’ academic self-efficacy (Gota, 2012). Evenly speaking, the findings of the present study shed light on the role of a new parent-related factor (i.e., living with single parents *per se*) in shaping students’ mathematics self-efficacy.

## 5. Conclusion, recommendations, and limitations

In the present study, it is evident that high school students in Addis Ababa have average mathematics self-efficacy. Given the critical role of students’ mathematics self-efficacy in their career choice and success, this could go against the country’s aspirations of attracting a large number of students to STEM fields.

The fact that educational expectations are important predictors of students’ MSE in Addis Ababa implied the ecological validity of Bandura’s social cognitive theory. Besides, the present study pinpointed the role of living arrangements with parents in predicting students’ mathematics self-efficacy, which calls for further investigations, preferably longitudinal with a large sample size and a wider scope, including additional variables (e.g., parenting style). Furthermore, researchers and organizations that are working with and/or concerned about students’ achievement, including but not limited to the Ministry of Education, need to gear their attention toward improving students’ mathematics self-efficacy and, thus, their future academic success and career choice.

Finally, the fact that this study includes a small sample size from only two schools, is conducted with only two grade levels, and is cross-sectional are the limitations of the study.

## Data availability statement

The original contributions presented in this study are included in the article/Supplementary material, further inquiries can be directed to the corresponding author.

## Ethics statement

The studies involving human participants were reviewed and approved by Addis Ababa University, School of Psychology review committee. The patients/participants provided their written informed consent to participate in this study.

## Author contributions

YY and SK conceived the study, developed the tool, and collected the data. YY analyzed the data. YY, AZ, and DG discussed the findings and developed the manuscript. All the authors read and approved the final manuscript.
