# Supplementary material for: Predictors of high school students’ mathematics self-efficacy in Addis Ababa: The importance of educational expectations
Source: Front Psychol. 2023 Jan 6;13:927757. doi: 10.3389/fpsyg.2022.927757 (PMC9852816; doi:10.3389/fpsyg.2022.927757)
Supplement: Supplementary file 1 [file Table_1.doc]

**The English version of the measure for MSE**

Here under is the English version of the questionnaire but bear in mind that the Amharic version is used in the study.

**Part II: Self-efficacy belief of students**

**Directions: Please use the following scale to respond to the following statements.**

**Read each statement carefully and answer as honestly as you can. You can select any number between 1 and 5. Please use the following rating scale.**

**The rating scale is as follows**:

1. Strongly disagree 2. Disagree 3. Undecided

4. Agree 5. Strongly agree

|  | **Items** | **1** | **2** | **3** | **4** | **5** |
| --- | --- | --- | --- | --- | --- | --- |
| 1 | It is important to me to get *good grades* in mathematics. |  |  |  |  |  |
| 2 | Compared to the ***boys in my math class***, I am good at mathematics. |  |  |  |  |  |
| 3 | Compared to the ***boys in my school***, I am good at mathematics. |  |  |  |  |  |
| 4 | Being good in mathematics is important to me. |  |  |  |  |  |
| 5 | Solving math problems is interesting to me. |  |  |  |  |  |
| 6 | Compared to the ***girls in my math class***, I am good at mathematics. |  |  |  |  |  |
| 7 | Compared to the ***girls in my school***, I am good at mathematics. |  |  |  |  |  |
| 8 | Compared to the ***all the students in my math class***, I am good at mathematics. |  |  |  |  |  |
| 9 | Compared to the other students my age, I am good at mathematics. |  |  |  |  |  |
| 10 | I get good grades in mathematics. |  |  |  |  |  |
| 11 | Work in mathematics classes is easy for me. |  |  |  |  |  |
| 12 | I’m hopeless when it comes to mathematics. |  |  |  |  |  |
| 13 | I learn things quickly in mathematics. |  |  |  |  |  |
| 14 | I have always done well in mathematics. |  |  |  |  |  |
| 15 | I find mathematics interesting. |  |  |  |  |  |
| 16 | When a math problem is difficult for me to solve, I just put more effort into solving it. |  |  |  |  |  |
| 17 | I will work as long as necessary to solve a difficult math problem. |  |  |  |  |  |
| 18 | When I find math homework difficult, I usually give up on it. |  |  |  |  |  |
| 19 | I enjoy doing mathematics homework. |  |  |  |  |  |
| 20 | Mathematics is boring. |  |  |  |  |  |
| 21 | I believe I could be a mathematician or a scientist when I grow up. |  |  |  |  |  |
| 22 | I have usually been at ease and relaxed during math tests. |  |  |  |  |  |
| 23 | Mathematics makes me feel uncomfortable and nervous. |  |  |  |  |  |
| 24 | I get really uptight during math tests. |  |  |  |  |  |
| 25 | I feel calm and relaxed when I work with mathematics |  |  |  |  |  |
| 26 | When I am taking math tests, I usually feel nervous and uneasy. |  |  |  |  |  |
| 27 | It does not scare me to take a math test. |  |  |  |  |  |
| 28 | I dread having to do math. |  |  |  |  |  |
| 29 | The thought of taking advanced high school math courses scares me. |  |  |  |  |  |
| 30 | When the teacher calls on me in class to answer a math question or solve a math problem, I worry that I will do poorly. |  |  |  |  |  |
| 31 | I find many mathematical problems interesting and challenging. |  |  |  |  |  |
| 32 | I have generally done better in mathematics courses than in other courses. |  |  |  |  |  |
| 33 | Mathematics makes me feel inadequate. |  |  |  |  |  |
| 34 | I am quite good at mathematics. |  |  |  |  |  |
| 35 | I have trouble understanding anything that is based upon mathematics. |  |  |  |  |  |
| 36 | I have always done well in mathematics classes. |  |  |  |  |  |
| 37 | I never do well on tests that require mathematical reasoning. |  |  |  |  |  |
| 38 | At school, my friends come to me for help in mathematics. |  |  |  |  |  |
| 39 | I have never been very excited about mathematics. |  |  |  |  |  |
